# Healthcare ecosystems research in mental health: a scoping review of methods to describe the context of local care delivery

**DOI:** 10.1186/s12913-019-4005-5

**Published:** 2019-03-18

**Authors:** Mary Anne Furst, Coralie Gandré, Cristina Romero López-Alberca, Luis Salvador-Carulla

**Affiliations:** 1 Centre for Mental Health Research, Research School of Population Health, ANU College of Health and Medicine, Australian National University, 63 Eggleston Rd Acton ACT, Canberra, 2601 Australia; 2 URC-Eco Ile-de-France, F-75004 Paris, France; 3 University Paris Diderot, Sorbonne Paris Cité, ECEVE, UMRS 1123, F-75010 Paris, France; 4 Inserm, ECEVE, U1123, F-75010 Paris, France; 5 Departament of Psychology, University of Cádiz, Cádiz, Spain

**Keywords:** Mental health care systems, Mental health care comparison, Mental health care delivery, Mental health systems research

## Abstract

**Background:**

Evidence from the context of local health ecosystems is highly relevant for research and policymaking to understand geographical variations in outcomes of health care delivery. In mental health systems, the analysis of context presents particular challenges related to their complexity and to methodological difficulties. Method guidelines and standard recommendations for conducting context analysis of local mental health care are urgently needed. This scoping study reviews current methods of context analysis in mental health systems to establish the parameters of research activity examining availability and capacity of care at the local level, and to identify any gaps in the literature.

**Methods:**

A scoping review based on a systematic search of key databases was conducted for the period 2005–2016. A systems dynamics/complexity approach was adopted, using a modified version of Tansella and Thornicroft’s matrix model of mental health care as the conceptual framework for our analysis.

**Results:**

The lack of a specific terminology in the area meant that from 10,911 titles identified at the initial search, only 46 papers met inclusion criteria. Of these, 21 had serious methodological limitations. Fifteen papers did not use any kind of formal framework, and five of those did not describe their method. Units of analysis varied widely and across different levels of the system. Six instruments to describe service availability and capacity were identified, of which three had been psychometrically validated. A limitation was the exclusion of grey literature from the review. However, the imprecise nature of the terminology, and high number of initial results, makes the inclusion of grey literature not feasible.

**Conclusion:**

We identified that, in spite of its relevance, context studies in mental health services is a very limited research area. Few validated instruments are available. Methodological limitations in many papers mean that the particular challenges of mental health systems research such as system complexity, data availability and terminological variability are generally poorly addressed, presenting a barrier to valid system comparison. The modified Thornicroft and Tansella matrix and related ecological production of care model provide the main model for research within the area of health care ecosystems.

## Background

The role of context is critical in health services research. Geographical variations in the fate of healthcare interventions have been documented widely. The significance of local context in such variations is recognised, with the more complex the intervention, the greater the relevance of local factors to its outcome [1]. In health care, “context” could be defined as all sources of evidence of the local system: geographic, social and demographic factors, other environmental factors, service availability and scope, capacity, use, costs and the historical development of the health care system. Evidence from the context of local health systems is thus highly relevant for research and policymakers. The analysis of context of care of “healthcare ecosystem research” is an emerging discipline that should play a critical role in implementation sciences [2] and in the analysis of complex interventions [1, 3]. However, a broader approach than the traditional unidimensional model of evidence is required [4]. “Contextual evidence” has recently been identified as a major source of knowledge in health systems research together with experimental, observational, expert and experiential knowledge [4]. In spite of its relevance, the need for context analysis in health services and delivery research has not been sufficiently recognised [1, 2, 4].

Evidence about local conditions is important at all stages in the policy process from assessing resource availability and setting policy priorities to examining the impact of policy decisions [5]. The World Health Organization (WHO) has urged exploration of the care context in mental health systems [6]. The WHO Mental Health Gap Action Program (mhGAP) has called for a comprehensive and systematic description of mental health services, including what those services are doing [6]. A knowledge of care delivery at the service delivery level is critical to evidence informed policy [7], and in the implementation of models of care such as integrated care [8] and the balanced care model [9]. However, this research faces challenges related to the complexity of mental health systems, and to methodological issues. Mental health care systems are particularly complex due to the number of sectors, levels, and types of service through which care is delivered, the variability of the service delivery over time and the high ambiguity, partly due to the lack of a stable terminology [1, 7]. Descriptions of local service delivery which do not take this complexity into account risk providing policymakers with an inaccurate or limited assessment of the local pattern of service availability, affecting their ability to plan appropriately.

A review of methods used to describe the context of local mental health care is urgently needed. This study sought to take a broad view of available methods of context analysis in systems of mental health care delivery at the service delivery level, identifying and mapping their main components and characteristics. This would identify gaps, provide insight into conceptualisation of the context of mental health systems and inform future context analysis in mental health services research. This is consistent with the call by the WHO to specifically reference service location, availability and function [6].

## Methods

### Rationale for conducting a scoping review

Scoping reviews “examine the extent, range and nature of research activity in a particular field, without necessarily delving into the literature in depth or attempting to assess its quality” [10]. They are used to “identify parameters and gaps in a body of literature” rather than “generat (ing) a conclusion related to the focussed question”, with “inclusion/exclusion … developed post-hoc”, and a broad research question rather than a “focussed research question with narrow parameters” [10]. A scoping review was considered appropriate for this study due to the broad scope of the research area, the diversity of study designs already known to the authors, and the absence of a definitive terminology.

### General scoping review process

We have used the five stage model for scoping reviews developed by Arksey and O’Malley [11], and extended by Levac [12]. The five stages of this approach are: (i) identifying the research question; (ii) identifying relevant studies; (iii) selecting studies; (iv) charting the data; and (v) collating, summarising, and reporting the results. We have also used the guidance for scoping reviews developed by members of the Joanna Briggs Institute [13].

### Identifying the research question

The main research question of this scoping review was:

1. “What are the main gaps in the available literature relevant for context analysis of mental health system*s*?”

Sub-questions are:

(i) “What are the available methods for standard description of mental health service delivery which could be applicable for international context analysis of mental health systems?”
(ii) “What are the key domains or components of methods for context analysis in mental health systems research?”

An additional objective of this scoping review was to identify a workable set of search terms that optimise the literature review in this new research area.

In order to answer these questions we have adopted a systems dynamics/complexity approach [14], and a modified version of Tansella and Thornicroft’s matrix model of mental health care (TT-Matrix) [15] (Table 1) as the conceptual framework for our scoping analysis. Tansella and Thornicroft developed this framework to facilitate the “bridging of information between different levels of analysis” [15], and to address issues related to system complexity encountered in mental health systems research: for example, the conflating of proxies of inputs or processes such as the number of psychiatric beds used, with outcome; and a failure to take account of evidence obtainable at different levels of the system through a reliance on experimental evidence gained at the individual or micro level [15]. The matrix concept has continued to be developed in mental health services research to provide a basis for mental health performance measurement [16, 17]. The modified version of the TT-Matrix (mTT-Matrix) provides 12 quadrants of indicators of health care according to the Donabedian process of care (input, throughput and output [18]); and the levels of care: 1) macro (country or region); 2) meso (local-catchment areas); 3) micro (facilities, services, care teams); and 4) nano (individual agents such as consumers, carers and professionals). We are looking specifically at the care service delivery system at the meso level (quadrant 2A), and the aggregation of information from the micro level to the meso level (quadrant 3A), and from meso level to macro level (quadrant 1A).

**Table 1:** Modified version of the Tansella-Thornicroft Matrix of Mental Health Care (mTT-Matrix)

	INPUT	THROUGHPUT	OUTPUT
Macro Country/Region	1A	1B	1C
Meso Local area	2A	2B	2C
*Micro Service* ^*a*^	*3A*	*3B*	*3C*
Nano Individual	4A	4B	4C

^a^ *The micro level at the original TT-Matrix referred to individual patients or consumers. In this modified version “Micro” refers to the process of care at the service level and “Nano” at the level of individual agents (users, peers, carers and professionals)*

### Identifying relevant studies

A systematic search was carried out, using the above research questions: the period of reference was 2005–2016. Databases used were the Cumulative Index to Nursing and Allied Health Literature (CINAHL), Web of Science (WoS) and Medline databases. LSC and MF selected the search terms. Broad terminology was required due to the low specificity of the applicable terminology. The search was carried out with the assistance of an academic librarian. The search terms used for the first search using CINAHL, WoS and Medline were (“mental health care” OR “mental health care delivery” OR “mental health service*” OR “mental health system*” OR “psychiatric service” OR “psychiatric care”) *AND* (classification OR description OR availability OR “meso-level analysis” OR “meso level analysis” OR “geographical mapping” OR mapping OR “healthcare instrument” OR “health care instrument” OR “healthcare tool” OR “health care tool” OR “local care”).

Some key articles known to the authors were noted to be missing, so an additional search was carried out using key words from those articles; these were mental health AND (“cross-country comparison*” OR “cross country comparison*” OR “international comparison*” OR “cross-cultural comparison*” OR “cross cultural comparison” OR “health system* research”). A search of the British Library on Demand database was also made using all the above key words. Further titles, by an author with an interest in the area, known to one of the authors (LSC) were added.

### Study selection

MF conducted the database search based on the search terms, and conducted a review of titles. Abstracts of potentially relevant papers were identified, and duplicates were deleted. Studies were initially included if they described or conceptualised the context of mental health care; mapping of mental health services; service availability, capacity or accessibility in geographic areas, or instruments assessing service availability, capacity or accessibility. Initial exclusion criteria were papers only reporting on service utilisation, interventions, financing and costs, and governance, due to their being not specifically related to availability. Also excluded as being too limited in scope were studies related to specific groups, such as child and adolescent mental health, mental health of culturally and linguistically diverse (CALD) populations, forensic mental health, or veterans’ mental health. Conference abstracts and non- scientific literature were excluded as their inclusion would have created an unfeasibly large database. Eligible study designs were broad, and included qualitative analysis gathered by experts, studies using a mixed approach, modelling studies, secondary analysis from databases, surveys and comparative studies. At this point we decided to include studies where the comparison was within countries and not just international or cross country, in case these methods could potentially also be used in cross country comparison.

The identified abstracts were reviewed by MF and CG, who discussed differences, and, where they could not be resolved, a further discussion was held with LSC. Study selection was an iterative process. In meetings with MF, CG and LSC, and due to increasing familiarity with the scope of papers, the search was refined, with additional exclusion criteria applied: papers reporting only on workforce or placement or bed capacity, or those including data exclusive to only one domain of care (residential, outpatient care, or day services), (unless describing all services in that domain) were excluded, again due to being too limited in scope. Micro level studies were also excluded as not relevant to the level of the system under study. It was noted that papers could be separated into conceptual, analytical and descriptive categories. At this point, a preliminary framework for data extraction was identified and piloted with five papers, based on the emerging picture of study characteristics.

The remaining full texts were read by MF and CG. Papers were excluded at this stage again for limited or incompatible interpretations of the concept of service availability, including service utilisation, service capacity only; or for providing no data on availability. A further number of grey literature articles were excluded. Conceptual papers were also excluded at this point as being outside the scope of the question, which related specifically to methods used. References of included papers were hand searched for further articles by MF and CG and cross checked in the same manner.

MF and CG then met again with LSC, and discussed the different categories of data to be extracted from the included papers.

### Charting the data

A data extraction tool was discussed, based on the characteristics of the included papers. It was piloted with five papers by LSC, MF and CG. MF and CG then each used the tool on all the included papers, following which they reviewed each others’ decisions. Differences were discussed, and any that could not be resolved were discussed with LSC for a final decision.

The data extraction tool categorised papers into descriptive and analytic studies. It then focussed on the key characteristics of the studies, and finally on the methods used in the mental health system descriptions. Extracted study characteristics were those describing the type or scope of services and included target population (specific target population such as people with lived experience of mental illness, carers, specific diagnostic group such as depression, eating disorders, etc.) and whether this was formally defined; socio-economic context if described; the sectors described (health, social, education, employment, housing, other); service types (hospitals, clinics and so forth); care branches (domains of service delivery); workforce capacity (types of professionals); placement capacity (beds or places where described), and geographic accessibility (distance to services for service users). Variables relating to the methods used included the framework (if the study used a standardised framework); study geographical boundary and whether this was formally defined; level of analysis (macro, meso, or micro as above); classification or taxonomy if included in framework; study design; and presence and type of comparison.

### Collating, summarising and reporting the results

We first performed a numerical analysis of the characteristics of the papers to provide an overall picture of the geographic and demographic characteristics of the studies, and basic methodology (whether or not a standardised framework was utilised). As the methods used to describe mental health care delivery included several instruments, we then created a table of key analytical characteristics of each instrument. All in all, six instruments were used in the studies (see Table 3). While the scientific literature included many papers using data obtained through these instruments, in several papers only data from selected sections of the particular instrument were used, or in the case of WHO Assessment Instrument for Mental Health Systems (WHO-AIMS) and the Mental Health Country Profile (MHCP), only selected extracts from the whole country report were included in the study. Therefore, where possible, the full characteristics of these tools have been gathered from the original report documentation to enable a full description of the instrument or framework. In the case of the Adult Service Mapping Exercise (ASME), we were not able to locate the core instrument online. Following this, we analysed the key conceptual approaches taken by the identified methods of context analysis of mental health care delivery.

## Results

### Search results

**Fig. 1:**
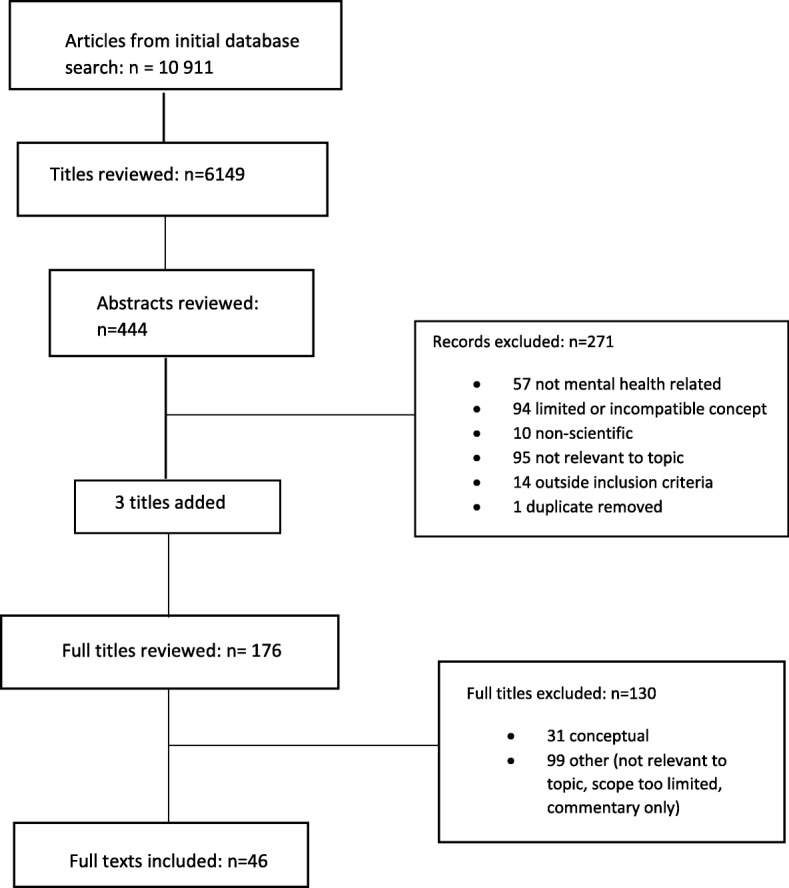
PRISMA flow chart of article selection

10,911 titles were identified in the initial search. After removal of duplicates, 6149 papers remained. After review of titles, 444 abstracts remained, following review of which 271 were excluded. Ninety-five were not relevant to the topic; 57 were not mental health related; 94 papers were excluded due to interpretations of the concept that were either limited (one type or branch of care only), or incompatible with the study concept (for example studies of service or resource utilisation, system governance, interventions, or care needs); 10 previously unidentified articles of grey literature were excluded at this point, as were 14 papers relating to areas of care outside the inclusion criteria, such as child and adolescent mental health, or CALD mental health. A previously unidentified duplicate was removed. Members of the team introduced three more papers for consideration based on knowledge of the scope of the study. The remaining 176 full text articles were reviewed independently by CG and MF: 130 papers were excluded either because they were not relevant to the topic; had a too limited scope (i.e. they related only to service capacity or to one type of service such as hospital acute care), or were commentaries and conceptual papers. All in all, 46 studies were eligible for inclusion in our scoping review (Fig. 1).

A shared meaning of key concepts in the assessment of mental health care delivery was lacking. For example, in full text papers reviewed, a number of papers were excluded where the concept of service availability had been variously interpreted as service utilisation, service workforce and service capacity. Thirty three papers related to service availability were excluded because they provided no data, 17 papers were excluded because they provided data only on workforce capacity, and seven papers were excluded because availability was conceptualized as either service utilisation or as availability of interventions.

#### Characteristics of included studies

Of the 46 eligible studies, 36 (78.3%) [19–54] were descriptive, and 10 (21.7%) were analytical [55–64]. Thirty six papers (80.4%) presented service availability data from a single country, of which 19 [20, 28, 32–34, 37, 40–42, 50, 51, 53–57, 59, 61, 64] took a regional or local approach, while 17 [19, 21–27, 30, 36, 38, 39, 43–46, 49] looked at availability from a national level. Ten papers presented service data from more than one country, of which seven [29, 35, 47, 48, 52, 58, 60] took a regional or local approach, and three [31, 62, 63] were at the national level. Overall, excluding two papers which included over 40 Lower Income Countries and Lower-Middle Income Countries (LIC/LMIC), not all of which were identified, 22 papers (48%) used data from Europe, most notably Spain and Italy, nine papers (20%) were from Africa, seven (15%) from Asia, four (9%) from the Middle East, two (4%) from the Americas (one from USA and one from Chile), and one (2%) from Australasia. Of the LIC/LMIC countries studied, eight were from Africa, and three were from Asia. However, in 25 studies (54.3%) the precise boundaries of the study area were not formally defined.

Twenty eight studies (60%) provided socio-demographic context [21, 24, 25, 27, 29, 31, 34, 37–40, 42–45, 47–50, 52, 53, 55–57, 59–62]. Two papers [34, 53] which presented data from atlases of mental health care included comprehensive local area data. Of the 16 studies which linked one or more socio-demographic indicators with mental health, only four provided supporting evidence with validated indicators using a standardised instrument (e.g. European Social Demographic Schedule -ESDS) [34, 48, 59, 60]. These four papers all used the European Service Mapping Schedule (ESMS) for service availability data. Papers based on WHO-AIMS and MHCP instruments also included legislative and policy context at a national level.

Where target populations were formally defined, 11 studies included children and/or adolescents [19, 21, 25, 30, 31, 36, 40, 50, 51, 53, 63]; three studies included people with alcohol and other drug dependence (AOD) [36, 61, 63]; two studies included people with intellectual disability (ID) [21, 36]; three were specific to serious mental illness or psychosis [57, 62, 63]; two included people over 65 years [21, 51]; and one study each included the following subpopulations: maternal/perinatal mental health [36]; people requiring long term rehabilitation [54]; survivors of suicide attempts [57];and socially marginalized groups [47]. A further 21 studies did not specify a particular mental health population.

The main characteristics of included studies are detailed in Table 2.

**Table 2:** Characteristics of included studies

Framework	Number (%) of total studies	Type of study		Location of study						Socio-dem ographic context provided	Study population				
		Descriptive	Analytical	International	Single country	Regional approach	Study area boundary formally defined	Includes Com-parison	LIC/LMIC*		Target population Formally defined	Adult MH only	Include at least one specific sub group**	Diagnosis specific	Mental health: population not specified
ESMS/DESDE	12(26.1%)	6	6	4	6	10	9	7	0	8	9	6	3	0	3
WHO-AIMS	11(23.9%)	9	2	3	9	9	7	4	7	6	2	0	4	3	4
MHCP	3(6.5%)	3	0	0	3	0	3	0	3	3	0	0	0	0	3
PRIME study	1(2.2%)	1	0	1	0	1	1	1	1	1	0	0	0	0	1
PROMO study	1(2.2%)	1	0	1	0	1	1	1	0	1	1	0	1	0	0
Adult Service Mapping Exercise	3(6.5%)	2	1	0	3	2	3	3	0	1	2	1	2	0	0
Method described	10(21.7%)	9	1	1	10	2	1	5	0	4	1	1	3	0	6
No method provided	5(10.9%)	5	0	0	5	4	0	0	0	3	0	0	1	0	4
	46 (100%)	36 (78%)	10 (21.7%)	10 (22%)	36 (78%)	22 (48%)	25 (54%)	21 (46%)	11 (24%)	27 (58.7%)	15 (32.6%)	8 (17%)	14 (30%)	3 (7%)	21 (46%)

*Low Income Countries/Low-Middle Income Countries, ** Eg child/adolescent; socially marginalised; older adults

We then analysed the methods used in included studies (Table 3). Six instruments providing data on service availability were identified in the included studies, and these were used in a total of 31 papers. Three of these were psychometrically validated instruments: ESMS/DESDE (Description and Evaluation of Services and Directories for Long Term Care-an evolution of the ESMS and thus described together) (used in 12 papers: [20, 28, 34, 48, 52, 53, 56, 58–61, 64]); WHO-AIMS: (used in 11 papers [21, 22, 24, 25, 27, 31, 33, 50, 51, 62, 63]); and MHCP (used in three papers [43–45]). ESMS/DESDE and WHO-AIMS are based on taxonomies of care (ESMS/DESDE on a hierarchical tree taxonomy), and DESDE has undergone formal ontological analysis [65]. The MHCP is structured into four domains relevant to policy, including context, resources, provision and outcomes. However, while the MHCP provided a taxonomy for mental health systems generally, it should be noted that the domains for health service delivery did not include any classification of service types. Two other instruments- those of the Best Practice In Promoting Mental Health In Socially Marginalized People In Europe study (PROMO) in 14 European capital cities [47] and the Programme for Improving Mental Health Care in five LMICs study (PRIME) [29] were designed specifically for those studies, and were included in one paper each. The ASME, used in three papers [23, 54, 55], was designed specifically for the English context. WHO-AIMS, MHCP, and the instruments from the PRIME and PROMO studies are instruments designed specifically for mental health services, while ESMS/DESDE and ASME have a broader health service application. ESMS/DESDE was developed for all long term care services. Fifteen studies did not use a structured framework [19, 26, 30, 32, 35–42, 46, 49, 57], of which five did not provide any method [37, 40–42, 46]. Four of these [37, 40, 41, 42] formed part of a group of seven papers in a special supplement related to a conference on mental health care in capital cities: however three of this seven papers were excluded from this study as they did not include any data on service availability.

**Table 3:** Characteristics of methods used by included studies

Framework		ESMS/DESDE	WHO-AIMS	MHCP	ASME	PRIME study instrument	PROMO study instrument	Other papers (number)
Ontology based		Yes	No	No	No	No	No	0
Taxonomy based		Yes	Yes	No	No	No	No	0
Psychometrically validated		Yes	Yes	Yes	No	No	No	0
Unit of analysis	Macro (Organ-isations)	No	Yes	Yes	Yes	Yes	No	14
	Meso (Services)	Yes	Yes	Yes	Yes	Yes	Yes	13
	Micro (Teams)	Yes	No	No	Yes	No	No	5
Number of comparison studies	Regional comparisons within a single country	4	1	0	2	0	0	0
	International comparisons at regional level	4	0	0	0	1	1	0
	International comparisons at national level	0	3	0	0	0	0	2
	Longitudinal comparisons	0	0	0	0	0	0	4
Glossary included		Yes	Yes	No	No	No	No	0
Data sources		Service providers	National level data from ministries, organ-isations etc; aggregated regional data where national data not available	Govt and other national level data sources	Local Implementation Teams	Govt and non govt reports, triangulated with local key co-ordinators	Service providers	X
Sectors^a^ included		H,S,E,Ed,Ho,O	H,S,E,Ed,Ho,O	H,S	H,S	H,S,Ho	H,S,E,Ho	X
Mental health specific or generic		Generic health	MH specific	MH specific	Generic health	MH specific	MH specific	X
Accessibility		Open Access but requires training	Open Access	Instrument itself unable to be accessed online	Unable to access online	Accessible online but specific to PRIME study	Study specific-Unable to access instrument online	X
Study design		Survey/interviews	Survey/interviews	Survey/interviews	Survey	Survey	Survey/interviews	X

^a^ *H-Health; S-Social; E-Employment; Ed-Education; Ho-Housing; O-Other*

In the case of ESMS/DESDE papers, the unit of analysis was care teams provided by individual services, aggregated at local level (2A in the mTT matrix), while in WHO-AIMS, ASME and MHCP papers, services data was aggregated at national level (1A in the mTT matrix). Of the 23 papers not using taxonomy based instruments (i.e all those papers not using ESMS/DESDE or WHO-AIMS), eight, including all three papers using the MHCP, counted services provided at a higher organisational level of care, such as psychiatric hospitals in a local area, along with individual services, such as day centres or mental health departments in larger organisations, thus conflating these different levels of care [30, 36, 37, 39, 40, 43–45]. In a further seven papers [23, 29, 35, 41, 42, 55, 57], including two of the three papers using the ASME, individual services were conflated in the same way with individual care teams (section 4A of the mTT matrix) such as crisis resolution teams, or assertive outreach teams.

Of the 15 papers which did not use a specific instrument to frame their analysis of service availability data, three [30, 36, 39] used internationally based frameworks, five [19, 26, 32, 49, 57] used a framework relevant specifically to the region in which the study took place, four [37, 40–42] categorised their data around service types but did not justify their categorisation or their choice of units of analysis, and three [35, 38, 46] did not specify any framework for their data on service availability. Of those studies using international frameworks, two [30, 36] were based on the Mental and Social Health Atlas of Saudi Arabia, which used the framework provided by the WHO Mental Health Atlas, while the third drew broadly on the WHO Mental Health Atlas, as well as recommendations from the 2001 WHO World Health Report to structure their findings [39]. Three studies described service availability according to the specific structure of the national system under study [19, 26, 49], while one described service availability based on a regionally prescribed framework of services required for the prevention of recurrent suicidal behaviour [57].

Terminology used to identify units of analysis varied widely, but only ESMS/DESDE and WHO-AIMS provide glossaries of terms used. MHCP studies included detailed qualitative data at the local level in order to ameliorate the effect of terminological variability on data interpretation. Terms used in papers for residential care included “psychiatric hospitals”, “supportive homes”, “crisis homes”, “safe homes”, “social rehabilitation centres”, “group homes”, “short and long term residential units”, “community based psychiatric inpatient units, respite, and community residential facilities” and those for non-residential care including “day hospitals” “psychiatric clinics”, “outpatient clinics”, “day centres”, “mental health dispensaries”, “mental health departments in social diseases prevention centres”, “day treatment facilities”, “fixed clinics”, “outpatient department”, “community mental health centres”, “sheltered workshops”, “day activity services”; “crisis resolution teams”, “assertive outreach teams”, “early intervention in psychosis team”, “home care nursing services”, and “mobile crisis teams”.

Data was obtained from sources at different levels of the health system. Studies using the ESMS and DESDE and the PROMO instrument take a bottom up approach, gathering data from providers at individual service level. WHO-AIMS takes a top down approach, the papers using this instrument collecting national data at a high level from sources such as heads of departments, universities, and professional boards. Where the instrument was used at a regional level, data was collected from similar sources at that level. In these studies however, the data is still interpreted through a national prism. Papers using the MHCP instrument and that of the PRIME study used both national and local sources, both methods combining national level data with qualitative data from the local level gathered from sources including professionals, clients, families and other stakeholders. The PRIME study is undertaken at district level, but uses a top-down approach, with data from administrative databases, key officials and service heads. Data for the ASME was gathered at a national level from Local Implementation Teams, although one paper [54] first identified relevant Trusts providing rehabilitation services using the ASME, and then went to the individual units to obtain data. In the 15 papers using other, non- framework based methods, existing administrative databases or literature were sourced, with four also using surveys sent to senior health or government officials [36, 38, 39, 57].

Seven studies included the health sector only [30, 32, 38, 39, 46, 62, 63]. Eighteen studies included the health and social sectors [19, 20, 22, 23, 26, 29, 31, 33, 45, 49, 50, 52, 54–56, 59, 61, 64]. This included papers using MHCP and ASME. At least one other sector, such as employment, education, justice, or housing was included in almost half of included studies (21 papers) [21, 24, 25, 27, 28, 34–37, 40–44, 47, 48, 51, 53, 57, 58, 60]. This included papers using ESMS/DESDE, WHO-AIMS, and those from the PRIME and PROMO study. The instrument of the PROMO study included several sectors, but for a limited target population (marginalised populations).

Of the 36 studies undertaken within a single country, seven [28, 51, 53–55, 59, 61] included comparison at regional or local level, and four included a comparison over time [19, 30, 32, 38]. All of the ten cross country studies included comparison of service availability: seven at regional or local level [29, 35, 47, 48, 52, 58, 60], and three at national level [31, 62, 63].

Forty-one papers (89%) identified themselves, or were assessed by us, as being situational and/or gap analyses. The remaining five papers comprised the following: efficiency analyses [58, 64] territorial planning [59], ecological analysis [57] and standard description for comparison [60]. Thirty-two studies (70%) included recommendations for policy makers related to service provision based on the findings. Visual tools were used in 12 papers (25%), four of which incorporated graphics issued by Geographical Information Systems. In three of these the visual tool presented data on service availability.

The methodological characteristics of included papers are summarised in Table 3.

In those papers using instruments to provide data on service availability, this was WHO-AIMS in 11 papers (24%) [21, 22, 24, 25, 27, 31, 33, 50, 51, 62, 63], ESMS/DESDE in 12 papers (26%) [20, 28, 34, 48, 52, 53, 56, 58–61, 64], MHCP in three papers [43–45] (7%), ASME in three papers (7%) [23, 54, 55] and the PRIME [29] and PROMO [47] project instruments in one paper each (2%).

## Discussion

To our knowledge this is the first scoping review on methods for context analysis of system provision and healthcare ecosystems research in mental health. Scoping reviews are appropriate in new areas of research, where they can “identify gaps in the research knowledge base, clarify key concepts, and report on the types of evidence that inform practice in the field” [13]. They “examine the extent, range and nature of research activity” [10]. Research questions are thus “less likely to address very specific research questions” but become more focussed in an iterative approach, due to the requirement that they identify all relevant literature regardless of design [11]. They are broad in nature to provide breadth of coverage: comprehensiveness and breadth are important in this search [12]. Thus, scoping studies may often produce very high numbers of initial results [10, 66, 67]. The lack of a clearly defined terminology, reflected in the wide range of search terms which needed to be included, reinforces the need for an approach taking a broad view of the literature. For these reasons, a scoping review was considered to be a more appropriate review method than a systematic review, which would require a focussed question with clearly defined outcomes.

### Implications for research

The WHO has called for description of systems of mental health care delivery and the gap analysis [6], but few standardised and validated methods are available to do so. Despite the complexity of mental health systems, many studies lack key methodological components such as a standardised framework, explanation of terminology, or explanation for choice of units of analysis: of the 46 papers included, 21 had serious methodological limitations, limiting their validity in international comparisons. The final number of included studies relative to the high number of initial results in the literature search indicates both a limited amount of research, and a lack of targeted and standardised research terminology in the area. The limited number of studies providing an explanation of the concepts or terms used presents difficulties when comparing systems, particularly across regions or countries, where the variation between systems may be greatest. The exclusion of full text papers due to limited interpretation of the concept of availability, or a conflation of availability with utilisation, demonstrates the lack of conceptual clarity in research in this area.

Comparisons between systems of care enable the sharing of knowledge, assist in problem solving and inform best practice. However, the replicability and comparability of several studies was undermined by a lack of clarity around terminology and scope, by the absence of structural organisation such as a taxonomy, and by inaccessibility or poor accessibility of some core instruments. A standardised framework was used in only half of those studies providing comparisons, and target populations were often either not specified (21 papers) or were very broad. The dearth of studies providing an explanation of the concepts or terms used was particularly relevant in comparisons across regions or countries, where the variation between systems may be greatest. Variation in terminology also creates a commensurability risk if units of analysis are not clearly defined and located within the overall system. The need for internationally agreed glossaries of terms has been underscored recently [68]. While the use of international frameworks enables international comparison, where the frameworks for data analysis are specific to a specific country or region, this is not the case. Lack of an analytical framework, or of a justification of the choice of units of analysis, limits the relevance of findings.

A systems thinking approach in health services research has been widely advocated [69]. Methods such as those of the ASME or MHCP which included only health or one other sector, may fail to identify information from other parts of the system key to an accurate analysis. Whole system analyses such as the Atlases of health described in two papers, taking into account the wider ecosystem in which healthcare operates, will be increasingly relevant to the emerging discipline of health ecosystems research.

Socio-demographic indicators varied, and were frequently not linked to evidence supporting their use in relation to need for mental health services. The level of availability of socio-demographic data was consistent with the level of availability of service delivery data presented in each article: i.e. national level socio-demographic characteristics where service delivery at a national level was reported. However, difficulty in obtaining relevant socio-demographic data was described in several papers, particularly those reporting studies carried out in LIC/ LMICs, or at lower than national level. This, and the identification of only one standardised instrument to collect such data suggests the need for a more systematic approach to the provision of socio-demographic context for assessment of service availability to be made within the context of local need.

Data aggregated at national level is not necessarily representative of the pattern of care across smaller areas, and may result in ecological fallacy. Additionally, administrative databases may be unreliable data sources, particularly in less resourced countries [70]. A bottom-up approach, gathering data at the local or regional level, can provide a more accurate and detailed picture of health care availability in small areas. However, local data can also be unreliable, difficult to obtain, and may not be collected routinely at local level. One paper identified in the search [71] related to the Emerging Mental Health Systems in LMICs’ (EMERALD) project. While it did not include data on availability, and was thus excluded from the study, it focused on capacity building for mental health research in these countries, and is thus critical in this area, particularly in the context of the relatively low number of studies identified from LIC/LMIC. Of the identified instruments using local sources of evidence, only two (ESMS/DESDE and MHCP) were standardised and psychometrically validated, and only one of these (ESMS/DESDE) gathered data on availability at this level, enabling its use in comparative studies.

### Implications for policy

Policy makers require evidence from the local context as well as global evidence at all stages of the policy making process to inform policy options [5]. Data on service availability and capacity using a whole system approach can help identify gaps or duplications in care delivery, enable comparison of best practice with other areas, and assist in the prediction and monitoring of the effect of interventions. However, the research to policy gap is well documented. Guidance for health systems which is “transparent, systematic and adapted to the local contexts…(and) …use (s) validated approaches.., in user-friendly formats” can bridge this gap [72]. Studies which use validated instruments and a bottom up approach, collaborating with local services and policy makers to identify local need, collect data and validate information gathered, are most likely to satisfy these criteria [34]. Interpretive aids such as visual tools and glossaries, which increase accessibility to complex data could also improve dissemination and policy uptake.

#### Limitations of the study

1/ LSC participated in the development of one of the tools which introduced potential bias. However, this was limited by the selection process being undertaken by MF and CGE, who were not involved with the development of the system, and at this time had no experience in the use of it.

2/ Grey literature was not included in this review. However, as stated, a very high number of results were returned by the search due to factors such as imprecise terminology in the area. Had grey literature also been included, the number of results could have threatened the feasibility of the review. In some cases, copyright restrictions or lack of availability of the core instrument meant we could not access the core instrument.

## Recommendation for future studies

The development of validated guidelines for the analysis of context of local service delivery is needed to increase the reliability of context studies and their relevance to policymakers through a more standardised approach. These should use a whole systems approach and provide standards for the description and grouping of target populations for international comparisons. They should also include interpretive aids such as glossaries to standardise terminology and key conceptual terms, as well as visual representations of complex data.

Further research is needed in LIC/LMIC to redress the current balance favouring Upper Income Countries in research. Developing capacity in LIC/LMIC through projects such as the EMERALD project, as well as standardised frameworks to enable comparison, is needed to enable this.

Future studies should ensure their core instrument is accessible for replicability. They should also systematically assess socio-economic context and formally define target population. There is a need for more analytical studies as opposed to purely descriptive papers.

## Conclusion

This scoping review has identified that context studies in mental health services is an area of limited research. Instruments with which to assess service availability are scarce, with some of those identified not easily accessible or unable to be generalised. Fifteen papers, or around one third of included studies did not use any kind of formal framework, and five of those made no description of method. Most studies presented a limited view of the system under study, even when using data collected by instruments designed to take a wider systems view. Four of the six instruments identified (ESMS/DESDE, WHO-AIMS, and the instruments of the PRIME and PROMO studies) took a whole system approach, but two of these (WHO-AIMS, PRIME) were from a top down perspective, and thus constrained by the limitations to local relevance of aggregated data. One instrument (ESMS/DESDE) is readily accessible and validated, and takes both a local approach and a whole systems perspective, and was used in 12 papers. In general, the challenges of commensurability, of terminological variability, and of data availability and validity which face this area of research are poorly addressed, with few standardised frameworks available and only three of these (ESMS/DESDE, WHO-AIMS, MHCP) having undergone psychometric testing. This presents a barrier to valid system comparison, particularly across regions or countries, where regional and historical variations in service provision increase terminological variability. On the other hand, we have identified the relevance to this area of research of use of a standardised instrument, formal geographic boundaries, a glossary of terms, formal target populations and a whole systems approach.

## Abbreviations

AOD: Alcohol and Other Drugs
ASME: Adult Service Mapping Exercise
CALD: Culturally and Linguistically Diverse
CINAHL: Cumulative Index to Nursing and Allied Health Literature
DESDE: Description and Evaluation of Services and Directories for Long Term Care
EMERALD: Emerging Mental Health Systems in Low and Middle Income Countries
ESDS: European Social Demographic Schedule
ESMS: European Service Mapping Schedule
ID: Intellectual Disability
LMICs: Low and Middle Income Countries
MHCP: Mental Health Country Profile
mhGAP: WHO Mental Health Gap Action Program
mTT: Modified Tansella and Thornicroft matrix
PRIME: Programme for Improving Mental Health CarE
PROMO: Best Practice In Promoting Mental Health In Socially Marginalized People In Europe
TT-Matrix: Tansella and Thornicroft’s matrix model of mental health care
WHO: World Health Organisation
WHO-AIMS: World Health Organisation Assessment Instrument for Mental Health Systems
WoS: Web of Science
